# The effects of greater frequency of two most prevalent bothersome acute respiratory symptoms on health-related quality of life in the 2020 US general population

**DOI:** 10.1007/s11136-022-03319-4

**Published:** 2023-03-11

**Authors:** John E. Ware, Graça Coutinho, Adam B. Smith, Evi Tselenti, Anuradha Kulasekaran

**Affiliations:** 1 John Ware Research Group, 10 Wheeler Court, Watertown, MA USA; 2 Tufts University School of Medicine, Boston, MA USA; 3 Global Medical Affairs Respiratory, Reckitt Health, Slough, UK; 4 Health Outcomes, Reckitt Health, Hull, UK

**Keywords:** Respiratory symptoms, Acute cough, Acute sore throat, Health-related quality of life, SF-36, SF-6D, Minimally important difference

## Abstract

**Purpose:**

Upper respiratory tract infections (URTI) and related symptoms are widespread and a common reason for visiting primary care with cough and sore throat being most prevalent. Despite their impact on daily activities, no studies have explored the impact on health-related quality of life (HRQOL) in representative general populations. We aimed to understand the short-term impact of the two most prevalent URTI symptoms on HRQOL.

**Methods:**

Online 2020 surveys including acute (≤ 4 weeks) respiratory symptoms (sore throat and cough) and SF-36^®^ health survey (all with 4-week recall) were analysed using analysis of covariance (ANCOVA) in comparison with adult US population norms. Linear T-score transformation of SF-6D utility (ranging from 0 to 1) enabled direct comparisons with SF-36.

**Results:**

In total, 7563 US adults responded (average age: 52 years; range: 18–100 years). Sore throat and cough lasting at least several days were experienced by 14% and 22% participants, respectively. Chronic respiratory conditions were reported by 22% of the sample. A clear and consistent pattern of group HRQOL means declining significantly (*p* < 0.001) for acute cough and sore throat symptom presence and severity. Declines were observed on SF-36 physical (PCS) and mental component (MCS) and health utility (SF-6D) scores controlling for covariates. Those reporting respiratory symptoms ‘most days’ declined ≥ 0.5 standard deviation (minimal important difference [MID]) worse with averages at the 19th and 34th centiles for cough on the PCS and MCS, and 21st to 26th centile for sore throat.

**Conclusion:**

Declines in HRQOL with acute cough and sore throat symptoms consistently exceeded MID standards and should not be ignored as self-limiting without intervention. Future studies on early self-care for symptom relief and its implications on HRQOL and health economics would be valuable to understand the benefits on healthcare burden and need for updating treatment guidelines.

**Supplementary Information:**

The online version contains supplementary material available at 10.1007/s11136-022-03319-4.

## Plain English summary

Acute cough and sore throat are common symptoms experienced by people every year from various causes. Day-to-day living is known to be affected by short-term (< 4 weeks) cough and sore throat symptoms but, to date, we have not been able to show by how much or the extent to which health-related quality of life can be altered. Our aims are to show that, even if these symptoms are self-limiting, we are able to measure their direct impact on emotional and physical quality of life, which can be applied to the general population. The results show that around one fifth of study participants were currently experiencing these acute symptoms. The impact from the presence of symptoms was significant—as both their emotional and physical health were affected, which could impact their day-to-day normal living. The worse their symptoms were or the longer they had them, the larger the impact on health-related quality of life. Although cough and sore throat symptoms are temporary, they have a significant impact on quality of life in the short term; this is worse for those who have other chronic respiratory conditions. These findings should encourage patients to start self-care early to relieve their symptoms and healthcare professionals to recommend the same, providing the necessary advice and treatments needed to manage the symptoms. These professionals should be cognizant of health-related quality of life in their routine practice and implement treatment guidance on self-care treatments.

## Introduction

Upper respiratory tract infections (URTIs), such as pharyngitis, laryngitis, rhinitis and nasopharyngitis, are the most common reason for attending a primary healthcare setting [1, 2]. URTIs, excluding coronavirus disease 2019 (COVID-19) symptoms, possessed the most substantial incident cases in 2019; although fatal consequences were relatively rare, morbidity was substantial and caused considerable disability-adjusted life years [1]. URTIs continue to represent a global threat and cause a significant societal impact both socially and economically, exceeding $22 billion for non-influenza-related infections [3, 4].

Seasonal viruses account for approximately 80% of acute respiratory infection cases, and last for about 8 to 10 days with a recurrence rate of at least twice a year in adults, while most children have approximately eight annual occurrences [5]. Although symptoms can vary, they typically start with sensation of sore throat, which may or may not develop into sore throat pain associated with nasopharyngitis, or tonsillitis or cough with or without fever [6, 7]. Symptoms associated with seasonal viruses can occur together; however, there is a tendency for differences in their onset of duration and persistence [8]. Acute cough tends to follow a sore throat and has been reported to last for a mean duration of 17.8 days, though this is reduced to 7.2 to 9.3 days in some specific scenarios; patients with chronic respiratory conditions such as asthma or chronic obstructive pulmonary disease (COPD) have a longer duration of this symptom [9]. While prevalence of sore throat and cough symptoms is at its peak during seasonal viral infections, there could be a low prevalence throughout the year from varied causes including and not limited to, environmental, physiological, or more recently fluctuating prevalence with COVID variants in the last few years [10–13].

Sore throat and cough are among the most prevalent and bothersome symptoms for patients with URTIs and a common reason for visiting primary care or seeking self-care treatments [8]. Cough tops the ten most common URTI symptoms and sore throat is one among these ten symptoms [2]. These symptoms may progress to the lower respiratory tract in those with airway deformities or comorbid conditions, manifesting as loss of smell and taste, difficulty in breathing, productive cough and shortness of breath. Although the management of acute sore throat [10] and cough is well described for symptomatic relief [4, 14] and includes medicated treatments with established efficacy and safety [15], there are innumerable non-medicated products with little to no information from robust efficacy studies [10, 14]. Overuse of antibiotics is an added serious and long-standing public health problem, particularly when half of this prescription is for URTIs [16], which are usually of viral aetiology, impacting negatively on quality-adjusted life years of patients from microbial resistance [17].

Historically, URTI impact on health-related quality of life (HRQOL), including physical functioning and mental health, was assessed in an acute care clinic setting [18]. However, URTI implications in the wider patient environment are not known. Availability of such information could transition the existing quality of care and treatment guidelines for early self-care given that HRQOL is well recognised by healthcare practitioners as an individual’s or group’s perceived physical, mental, and social functioning and well-being over time [19, 20]. Furthermore, there is an awareness that measuring HRQOL has benefits in terms of understanding symptom impact beyond what is typically believed to be a self-limiting condition. To our knowledge, there have been no previous attempts to quantitatively measure the short-term HRQOL impact of bothersome acute respiratory symptoms that is truly representative of impact that occurs naturally in the general population.

The primary aim of this study, therefore, was to demonstrate the association between the severity of bothersome acute respiratory symptoms on short-term HRQOL in the United States (US) general population. For the purposes of this report, we focus on the two most prevalent [2] and bothersome URTI symptoms—sore throat and cough—and seek to quantify their impact on daily HRQOL.

## Methods

### Study population and sample

Adults 18 years and older were drawn from National Opinion Research Center (NORC) AmeriSpeak^®^ true probability address-based panel. Participants were randomly selected to provide sample coverage of approximately 97% of the US household population (*N* = 1648) and a much larger (*N* = 5915) opt-in supplemental sample. As described in detail elsewhere [21, 22], this blended approach to sampling has the advantage of more cost-effectively estimating symptom prevalence and other population norms and calibration weights used in aggregating the probability sample with a larger opt-in sample, which increases the reliability of HRQOL impact estimates for those reporting the less-frequent, most severe acute severity levels [22]. Following pre-tests of survey length and programming, the online survey was administered to participants on the internet (98.6%) or by telephone (1.3%) over a 3-month period from April to July 2020. All participants provided informed consent prior to survey. The survey was conducted in accordance with the guidelines of the American Association for Public Opinion Research (AAPOR) and was approved by the NORC Institutional Review Board (protocol number 20.05.29).

### Survey

In addition to sociodemographic variables and the presence of a comorbid chronic respiratory condition (asthma and/or COPD), known to lower HRQOL [23] and increase the prevalence of respiratory symptoms, both sampling groups (probability sample and opt-in sample) were administered the same survey modules in the following order: new generic QGEN^®^ items under development [24] (not analysed here); standardised checklist for comorbid conditions [25]; SF-36 HRQOL Survey [23]; ratings of health now versus 3 months ago; acute respiratory symptoms; and impact of COVID-19 outbreak on personal life (not included as covariate). In addition to the above 94 items, as many as 72 additional items (median = 6) measured the HRQOL impact attributed to each chronic respiratory condition present.

### SF-36

The SF-36v2 was administered to assess HRQOL [26]. The SF-36v2 is a 36-item measure of a profile of eight domain scales, which are aggregated to estimate physical (PCS) and mental (MCS) summaries. The PCS and MCS are norm-based scores based on the US general population scores and converted to T-scores (mean of 50 and standard deviation [SD] of 10) to aid interpretation [27]. In addition, to compare the results with the overall health utility index developed for health economic evaluations, responses to SF-36v2 items were converted to SF-6D utility values ranging from 0 to 1 using standardised scoring methods [28] shown to represent societal preferences for health states where 1 reflects ‘perfect health’. In addition, a linear T-score transformation of SF-6D utility scores was performed to enable direct comparisons with physical and mental health component scores.

### Respiratory symptoms

Analyses focused on two acute respiratory symptoms (‘coughing’ and ‘sore throat’). Other respiratory symptoms surveyed included ‘loss of taste or smell’ and ‘shortness of breath or difficulty breathing’. Briefly, respondents were asked, “Over the past 4 weeks, how often have you been bothered by any of the following symptoms” according to a 4-point frequency scale (category 1 = ‘not at all’; category 2 = ‘several days’; category 3 = ‘most of the days’; category 4 = ‘nearly every day’) with an additional category 5 = ‘don’t know’ (not included in analysis due to low sample size). These symptom frequency response categories were adapted from surveys of prevalent respiratory symptoms [29].

### Statistical analysis

Categorical variables of interest were summarised descriptively by counts and percentages per category; severity was defined as the persistence of symptoms more often over days. Continuous variables were described using means and standard deviations. Patients were segregated as those with and without a chronic respiratory condition (asthma and/or COPD).

The relationship between each symptom and HRQOL was evaluated using analysis of variance (ANOVA) models. HRQOL differences equal to or greater than 0.5 standard deviation were accepted as satisfying the minimal important difference (MID) standard [30].

To confirm that acute respiratory symptom effects on HRQOL are generalisable, analyses of covariance (ANCOVA) controlled for the following sociodemographic variables (holdout groups are italicised): sex (*male*/female); age (four categories: 18–29, *30–44*, 45–59, 60 years and over); ethnicity (*white*, non-Hispanic; black, non-Hispanic; Hispanic; other); educational status (no High School diploma, *High School diploma*, some college, Bachelor’s degree or above); employment status (*working—paid employee or self-employed*; retired; not working—disabled; not working—other; not working—looking for work or temporary lay-off from work); income (< $29,999; *$30,000–$59,999*; $60,000–$99,999; > $100,000).

To control for the main effect of a chronic respiratory condition, which could increase acute respiratory symptom prevalence and/or lower HRQOL, the ANCOVA models included dummy variables for a chronic respiratory condition [25]. An interaction term (1 if both ‘Symptom every day’ and chronic respiratory condition reported, 0 otherwise) was added to the ANCOVA models to formally test for an interaction for both respiratory symptoms and all HRQOL outcomes.

Relative validity (RV) estimates were used to compare the ANOVA results within each symptom across the three HRQOL measures (PCS, MCS, and SF-6D). As in previous studies for this purpose [31], RV was calculated as the ratio between F-ratios for each HRQOL outcome in the ANOVA using the PCS as the referent value. RV < 1 indicated how much the PCS was more responsive relative to the comparators; values > 1 indicated how much the comparator was more responsive.

Data were imported and analysed using R (v4.0.3) [32]. A *p* value of ≤ 0.05, 2-tailed test, was considered statistically significant.

## Results

A total of 7563 US adults responded to the survey. Missing data were low (0.6% to 1.4%) for the overall items administered. The two samples had very similar characteristics including range of ages (18 to 100 years) although the probability sample (AmeriSpeak) averaged 6 years younger and were more nearly equal in gender than the Opt-In sample (Table 1). Approximately 50% of participants were between ages 30 and 59 years, with almost three quarters of the total sample participants being females. Overall, almost half were married (47%), had a bachelor’s degree or higher (54%) and worked in paid employment (44.3%). Similar percentages reported one or both chronic respiratory conditions across the probability (19.7%) and supplemental (18.1%) samples.

**Table 1:** Baseline characteristics of respondents in two samples

	AmeriSpeak panel probability sample (*N* = 1648)	Opt-in supplemental sample (*N* = 5915)
Mean age, years (SD)	47.17 (17.33)	53.23 (15.25)
Gender, *N* (%)		
Female	838 (50.9)	4805 (81.3)
Male	810 (49.2)	1110 (18.8)
Relationship, *N* (%)		
Divorced	180 (11)	872 (14.8)
Living with partner	153 (9.3)	344 (5.9)
Married	783 (47.6)	2778 (47)
Never married	435 (26.4)	1416 (24)
Separated	30 (1.9)	124 (2.1)
Widowed	67 (4.1)	381 (6.5)
Employment status, *N* (%)		
Working—paid employee	902 (54.8)	2448 (41.4)
Not working—retired	270 (16.4)	1831 (31)
Working—self-employed	145 (8.8)	361 (6.2)
Not working—other	113 (6.9)	346 (5.9)
Not working—disabled	107 (6.5)	299 (5.1)
Not working—looking for work	98 (6)	380 (6.5)
Not working—on temporary lay-off from a job	13 (0.8)	250 (4.3)
Respiratory comorbidities, *N* (%)		
Asthma	241 (14.7)	785 (13.4)
COPD	81 (5.0)	271 (4.7)
Chronic respiratory conditions^a^	80 (4.9)	268 (4.6)
Education, *N* (%)		
Bachelor's or above	558 (33.9)	3521 (59.6)
High School graduate or equivalent	339 (20.6)	754 (12.8)
Some college	669 (40.6)	1397 (23.7)
No high school diploma	82 (5)	243 (4.2)

*SD*  standard deviation, *COPD*  chronic obstructive pulmonary disease

^a^Asthma and COPD

### Distribution of symptom frequency across acute respiratory symptoms

In total, 77.8% (*N* = 5846) reported no coughing and 86.4% (*N* = 6460) reported no sore throat symptoms (Table 2). Similar patterns were also observed for the other 3 symptoms (loss of taste/smell, shortness of breath, fever) (Supplementary Table 1). In terms of the individual frequency by symptoms, 1910 (25.5%) reported experiencing at least one of the two symptoms and a small minority reported experiencing both symptoms on most or nearly every day (categories 3 and 4, respectively) in the last 4 weeks (*N* = 281; 3.8%) (Supplementary Table 2).

**Table 2:** Comparisons of HRQOL means (SD) across symptom frequency

Symptom and HRQOL measure (mean, SD)	Response category				*F* ratio^a^	RV
	Not at all	Several days	Most of the days	Nearly every day		
Cough	*N* = 5846	*N* = 939	*N* = 415	*N* = 312		
PCS-SF-36	51.9 (9.37)	45.9 (9.45)	42.1 (8.19)	41.7 (11.06)	313.27	–^b^
MCS-SF-36	51.1 (9.81)	45.6 (10.01)	41.5 (8.43)	42.4 (10.59)	250.72	0.8
SF-6D^c^	52 (9.36)	44.7 (8.63)	40.2 (6.88)	41.1 (10.8)	439.01	1.4
SF-6D	0.7759 (0.1554)	0.6539 (0.1433)	0.5806 (0.1143)	0.5954 (0.1794)		

Sore throat	*N* = 6460	*N* = 584	*N* = 290	*N* = 143		
PCS-SF-36	51.2 (9.71)	45.3 (9.52)	41.9 (7.03)	42 (10.82)	178.09	–
MCS-SF-36	50.8 (9.92)	42.8 (9.42)	39.3 (6.03)	39.6 (7.91)	289.02	1.62
SF-6D^c^	51.4 (9.47)	42.7 (7.92)	37.9 (5.85)	38.8 (10.66)	398.18	2.23
SF-6D	0.7664 (0.1573)	0.6208 (0.1315)	0.5411 (0.0972)	0.5573 (0.1770)		

*MCS* mental component summary, *N* = number of participants, *PCS* physical component summary, *RV*  relative variance, *SD*  standard deviation, *SF-36*  short-form 36, *SF-6D* six-dimensional health state short form

^a^All ANOVA results were statistically significant at *p* < 0.05; linear *T*-score transformation of SF-6D to enable standardised (mean = 50, SD = 10) direct comparisons with PCS and MCS

^b^Reference category for RV

^c^Linear T-score transformation of SF-6D

For each of the respiratory symptoms, prevalence declined with increased symptom frequency (‘several’, ‘most’ or ‘nearly every day’; Supplementary Table 2). Most prevalent (reported for at least several days in the past 4 weeks) was coughing (22.1%) and sore throat (13.6%). Similar trends were seen with other respiratory symptoms (Supplementary Table 2).

### HRQOL decline associated with greater symptom frequency

**Fig. 1:**
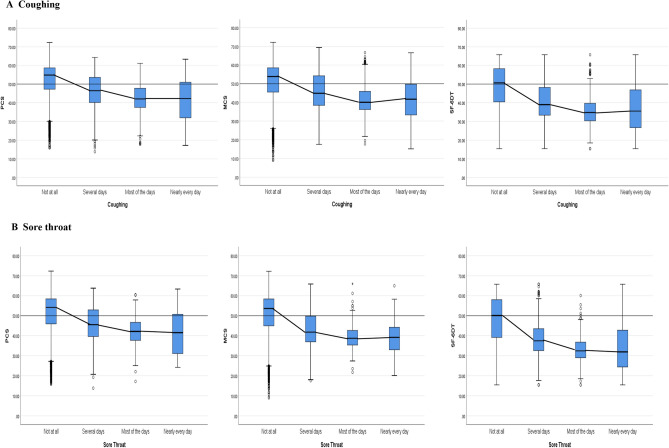
Health-related quality of life scores according to frequency of symptom responses for cough (**A**) and sore throat (**B**). HRQOL scores for PCS, MCS and standardised utility for the two examined respiratory symptoms (cough [**A**] and sore throat [**B**]) according to frequency experienced. For all symptoms assessed, HRQOL scores declined with increased frequency as experienced by the participant. *Note: HRQOL*  health-related quality of life, *MCS*  mental component score, *PCS*  physical component score

Mean HRQOL scores across symptom frequency categories for two respiratory symptoms are shown for the total sample in Table 2. There were statistically significant differences for both cough and sore throat. Overall, means for participants reporting no symptom (category 1) were above population means and declined substantially (> 0.5 SD) in comparison with means for the other severity categories 2 to 4, for each of the three (PCS, MCS & SF-6D Utility) HRQOL outcomes (Table 2 and Fig. 1). Further, declines are apparent on average, going from category 2 (‘several days’) to category 3 (‘most days’), but remained the same as 3 or worse for category 4 (‘nearly every day’).

The above patterns of decline in HRQOL with greater acute respiratory symptom frequency remained statistically significant with ANCOVA control for sociodemographic differences (*p* < 0.001) and the main effects of a chronic respiratory condition (decline of 0.55–0.65 SD).

### Relative validity: responsiveness of psychometric and health utility scores

The RV comparisons across the three HRQOL methods are shown in Table 2. Although both PCS and MCS declined with increased frequency for both symptoms, RV comparisons showed that MCS was substantially (1.6 times) more responsive than the PCS for sore throat but was less responsive for cough. In contrast, utility RV estimates showed that it was more responsive than both psychometric measures across both respiratory symptoms.

## Discussion

The aim of this study was to evaluate the relationship between HRQOL and the two most prevalent and bothersome acute respiratory conditions, sore throat and cough. The results demonstrated a consistent pattern of HRQOL declining in the presence of an acute respiratory symptom. This overall pattern of results was observed for the SF-36v2 physical (PCS) and mental (MCS) components, as well as the SF-6D. These results held when sociodemographic covariates including chronic respiratory conditions were also controlled for. These findings highlight that the presence of acute sore throat and cough significantly and broadly worsen HRQOL in the short term.

The magnitude of HRQOL declines (Table 2) in our general population survey potentially highlights the clinical importance of symptom effects on HRQOL for early self-care needs. Healthcare professionals may perceive the acute sore throat and cough symptoms experienced during an URTI to be relatively inconsequential. Previous studies have suggested that physicians believe patients seek care for alleviation of acute respiratory symptoms unnecessarily [33]. However, the results of the present general population survey indicate that suffering from these symptoms can significantly impact HRQOL and that the more days of symptoms experienced—they are more often bothersome—the more notable the impact on HRQOL. The findings support previous national population data obtained in 2010 and highlight the significant burden of respiratory symptoms [25].

To our knowledge, this is the first report comparing outcomes using SF-36v2 PCS and MCS, as well as the SF-6D utility index for acute respiratory symptoms in the same study. The same overall pattern of decline in HRQOL was observed across all three methods (PCS, MCS & Utility). However, that said, the results showed greater responsiveness of the MCS for sore throat compared with the PCS; on the other hand, the PCS measure showed greater responsiveness for cough, even though these acute URTI symptoms are principally physical in nature. The SF-6D utility showed greater responsiveness to both respiratory symptoms compared with either component score when analysed separately. This lends greatly to the argument that HRQOL is predicated on both physical and mental health components [28].

In addition to this, the potential advantage of greater responsiveness when both physical and mental HRQOL are affected, the utility values enable economic evaluations in terms of quality-adjusted life year comparisons of outcomes [28]. Similar results have also been described for other respiratory symptom-based conditions, such as allergies [34] and chronic rhinosinusitis [35], which have been shown to cause significant disruptions to daily living as well as impact productivity. These examples, together with studies that have highlighted the financial cost of acute URTIs [10, 36, 37], illustrate how the impact on individual and population HRQOL—and in particular the mental health component for sore throat, as demonstrated in this study—directly translates to tangible societal and economical burdens.

The mean differences observed (Table 2) with the presence and increased frequency of symptoms equalled or exceeded the predefined minimal important difference [38]. In this survey, the magnitude (≥ 0.5 SD) of the impact on HRQOL in the short term is similar to the declines reported in systematic reviews and meta-analyses of people suffering from chronic diseases [23, 38, 39]. In these studies, a decline of ≥ 0.5 SD in HRQOL is the threshold effect size of discrimination for changes in HRQOL. Thus, our results on effect size due to symptom presence and greater severity indicate that patients with acute sore throat and cough experience a decline in their HRQOL, albeit in the short term, which is clinically meaningful. Although this survey did not evaluate improvements from management of symptoms, the significant decline in HRQOL from no symptoms to several days (category 1 to 2) is sufficient to imply that management of these symptoms would be expected to improve HRQOL. There are data in the literature on acute cough to suggest that this is indeed the case [40]. For example, a significant mean improvement in HRQOL scores of 1.2 (*p* = 0.04) in the Leicester Cough Questionnaire (LCQ- acute) was noted in patients with acute cough treated with over-the-counter medicine versus placebo [40]. Further research is still required to confirm the benefits of early established over-the-counter self-care medicines for sore throat such as locally applied non-steroidal anti-inflammatory drugs (NSAIDs) and anaesthetics versus other remedies [41–43] and cough [14] compared with non-medicines on HRQOL and the subsequent impact on societal and economic burden.

One potential limitation of this study was the fact that it relied on self-reported symptoms of acute URTI. That said, the large survey data were obtained from both a true probability US population sample and an Opt-in sample. Symptom category response percentages were consistent across samples (see Supplementary Tables 1 and 2) and prevalence estimates were in the ballpark with those shown in previous studies [23]. Thus, combined probability and opt-in sampling strengthen generalisability and precision of the study results. Furthermore, overall response rates to the survey were high, with low rates of missing data, and all potential confounding variables were accounted for in the analysis.

Our results are important findings for patients, physicians and pharmacists. We clearly show that suffering from cough and sore throat for as little as one day can have a major impact on an individual’s HRQOL, even when other variables are controlled for. The widespread occurrence of acute sore throat and cough means that the potential impact of these symptoms on healthcare systems is significant. Moreover, work productivity and absenteeism are likely to be affected. Routine collection of HRQOL in patients with URTI should be considered to understand the true impact of these symptoms on individual’s lives and to enable appropriate care. In most cases, acute cough and sore throat are self-limiting and early self-care treatment recommendations may support patients in managing their symptoms directly. We demonstrate that the short form tools (psychometric and utility) can adequately detect the HRQOL burden of cough and sore throat. Potential use of this within a community healthcare setting could enable better patient–healthcare professional interactions that both support improvement in patient HRQOL and that help to reduce pressures on overburdened healthcare systems.

## Supplementary Information

Below is the link to the electronic supplementary material.

Supplementary file1 (DOCX 724 kb)
