# Supplementary material for: The effects of greater frequency of two most prevalent bothersome acute respiratory symptoms on health-related quality of life in the 2020 US general population
Source: Qual Life Res. 2023 Mar 11;32(4):1043–51. doi: 10.1007/s11136-022-03319-4 (PMC10007648; doi:10.1007/s11136-022-03319-4)
Supplement: Supplementary file 1 — Supplementary file1 (DOCX 724 kb) [file 11136_2022_3319_MOESM1_ESM.docx]

**Title:** The Effects of Greater Frequency of Two Most Prevalent Bothersome Acute Respiratory Symptoms on Health-related Quality of Life in the 2020 US General Population

# Supplementary data

*Supplementary Table 1.* *Comparisons of HRQOL means (SD) across other symptom frequency*

| **Symptom and HRQOL measure (mean, SD)** | **Response category** | | | | **F-ratio*** | **RV** |
| --- | --- | --- | --- | --- | --- | --- |
|  | **Not at all** | **Several days** | **Most of the days** | **Nearly every day** |  |  |
| **Loss of taste/smell** | **N=6706** | **N=306** | **N=293** | **N=179** |  |  |
| PCS-SF36 | 51.0 (9.81) | 43.7 (7.55) | 43.1 (6.80) | 41.3 (10.8) | 166.38 | -^†^ |
| MCS-SF36 | 50.7 (9.95) | 39.8 (7.62) | 39.2 (5.92) | 39.8 (8.10) | 305.95 | 1.8 |
| SF-6D^‡^ | 52.2 (9.99) | 35.75 (10.04) | 31.67 (10.05) | 39.68 (9.98) |  |  |
| SF-6D | 0.7633 (0.1575) | 0.5733 (0.1094) | 0.553 (0.0958) | 0.5536 (0.1695) | 390.05 | 2.3 |
| **Shortness of breath** | **N=6178** | **N=695** | **N=376** | **N=235** |  |  |
| PCS-SF36 | 52.0 (9.06) | 43.3 (9.04) | 40.0 (8.36) | 39.0 (11.27) | 492.57 | -^†^ |
| MCS-SF36 | 51.3 (9.57) | 42.9 (10.12) | 39.58 (7.91) | 39.8 (9.49) | 399.67 | 0.8 |
| SF-6D^‡^ | 53.18 (10) | 39.9 (10.03) | 32.13 (10.05) | 39.53 (9.99) |  |  |
| SF-6D | 0.7769 (0.152) | 0.6054 (0.1224) | 0.5442 (0.1033) | 0.5575 (0.1634) | 645.01 | 1.3 |

*All ANOVA results were statistically significant at p < 0.05. ^†^Reference category for RV. ^‡^standardised SF-6D.

MCS = mental component summary; N = number of participants; PCS = physical component summary; RV = relative variance; SD = standard deviation; SF-36 = short-form 36; SF-6D = six-dimensional health state short form.

## Supplementary Table 2. Health and Well-being Affected by Bothersomeness of Acute Respiratory Symptoms as per Response Categories*

| **Question** | **Symptom** | **Response category** | **N** | **%** |
| --- | --- | --- | --- | --- |
| \| Over the past 4 weeks, how often have you been bothered by any of the following symptoms? \| \| --- \| | Coughing | Not at all | 5872 | 77.8 |
|  |  | Several days | 941 | 12.5 |
|  |  | Most of the days | 417 | 5.5 |
|  |  | Nearly every day | 313 | 4.1 |
|  | Sore throat | Not at all | 6490 | 86.4 |
|  |  | Several days | 585 | 7.8 |
|  |  | Most of the days | 290 | 3.9 |
|  |  | Nearly every day | 144 | 1.9 |
|  | Loss of taste / smell | Not at all | 6707 | 89.6 |
|  |  | Several days | 306 | 4.1 |
|  |  | Most of the days | 293 | 3.9 |
|  |  | Nearly every day | 179 | 2.4 |
|  | Shortness of breath | Not at all | 6178 | 82.5 |
|  |  | Several days | 695 | 9.3 |
|  |  | Most of the days | 376 | 5.0 |
|  |  | Nearly every day | 235 | 3.1 |

*Frequencies for the SF36v2 PCS/MCS

## Supplementary Table 3. Health-related quality of life differences with increasing number of respiratory symptoms

| **Number of symptoms** | **PCS-SF36**  **(Mean [SD])** | **MCS-SF36**  **(Mean [SD])** | **SF-6D**  **(Mean [SD])** |
| --- | --- | --- | --- |
| **0 (no symptoms)** | 52.78 (8.76) | 51.95 (9.35) | 0.8 (0.15) |
| **1 (Any one symptom)** | 47 (10.53) | 47.92 (10.48) | 0.7 (0.15) |
| **2 (Any two symptoms)** | 42.48 (10.65) | 44.66 (10.95) | 0.64 (0.15) |
| **3 (Any Three Symptoms)** | 42.99 (10.1) | 43.16 (10.01) | 0.61 (0.15) |
| **4 (Any four symptoms)** | 42.74 (7.76) | 38.95 (7.4) | 0.56 (0.11) |
| **5 (Any five symptoms)** | 42.4 (8.21) | 38.93 (5.86) | 0.55 (0.13) |
|  | F (5,7516) = 297.48,  P<0.0001 | F (5,7516) = 293.11,  P<0.0001 | F (5,7387) = 469.39,  P<0.0001 |

MCS = mental component score; PCS = physical component score; SD = standard deviation; SF-36 = short-form 36; SF-6D = six-dimensional health state short form.

## Supplementary Figure 1. Health-related quality of life scores according to frequency of symptom response for shortness of breath (A) and loss of taste/smell (B)

**A. Shortness of breath**


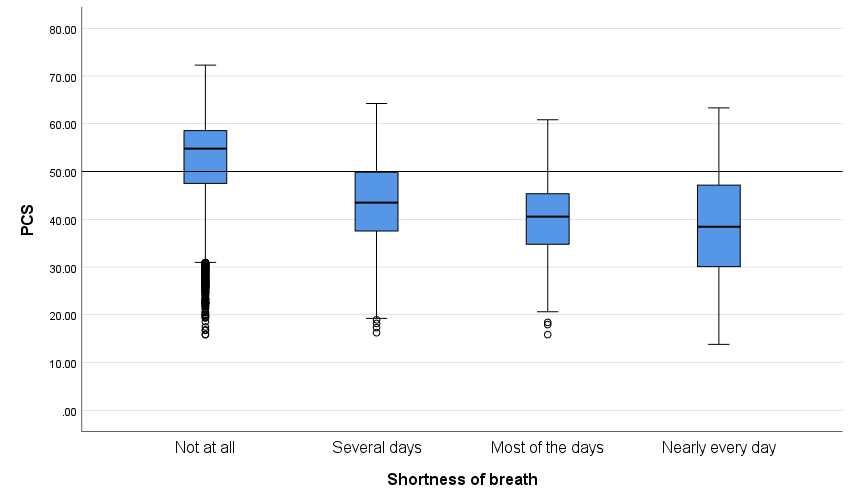

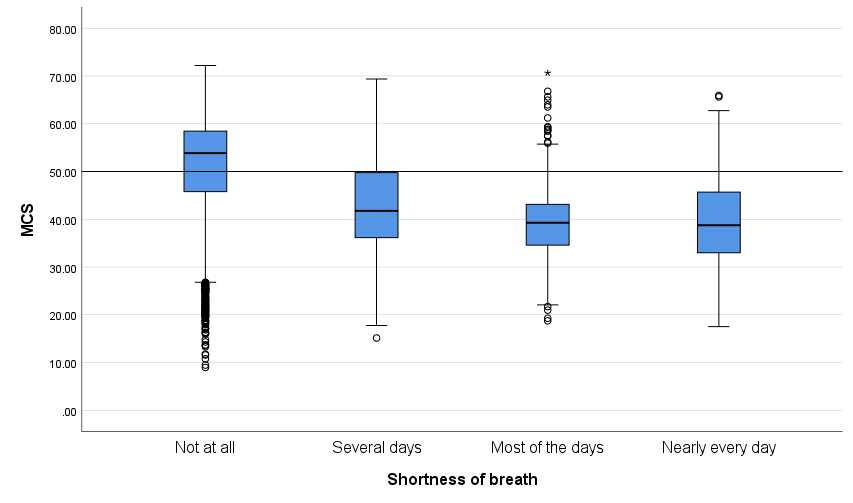

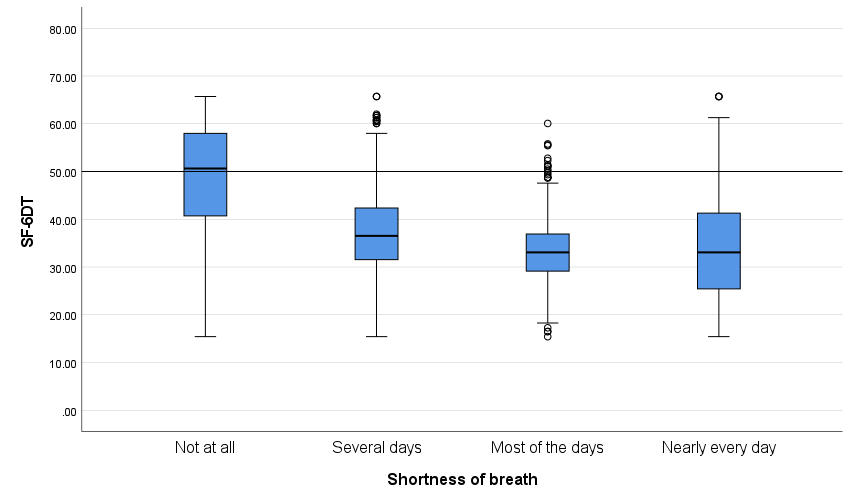


**B. Loss of taste / smell**


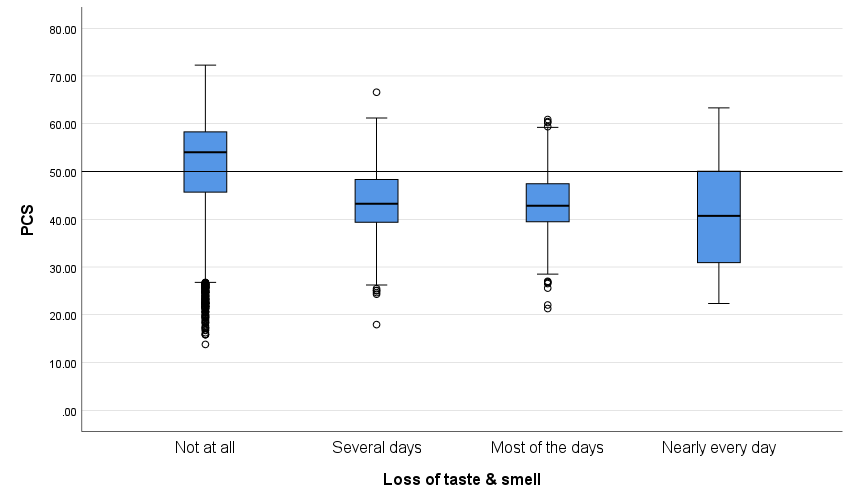

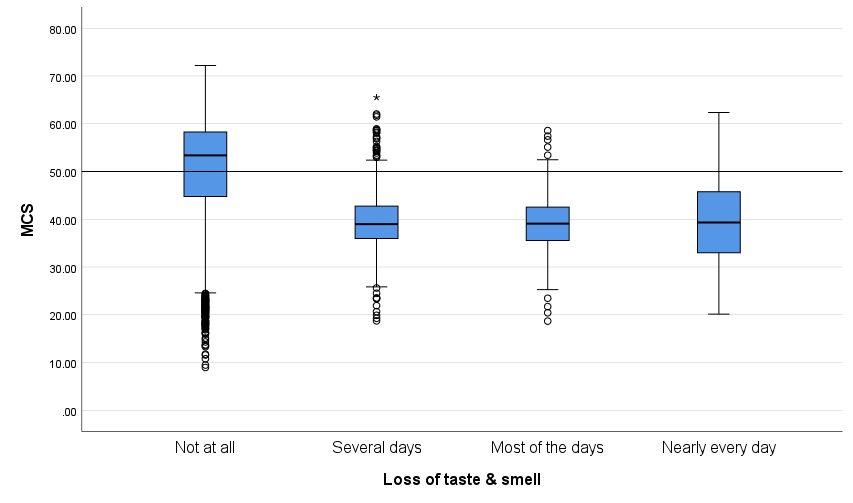

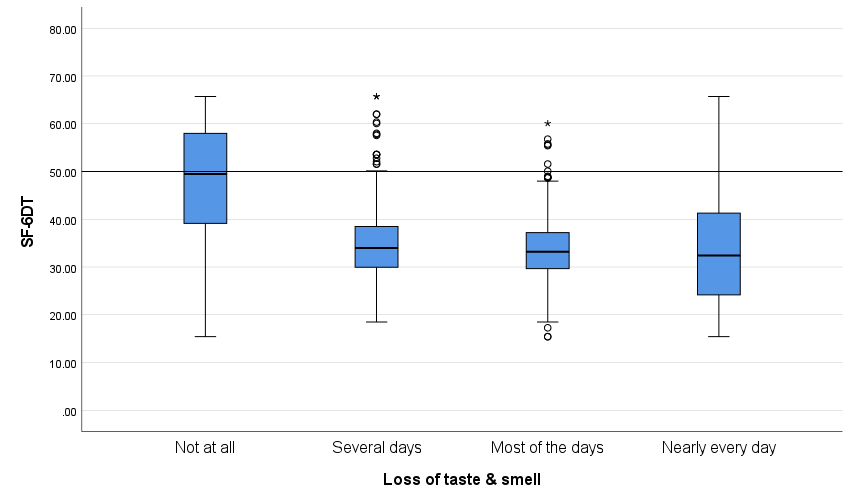


Supplementary Figure 1. HRQOL scores for PCS, MCS and standardised utility for the examined respiratory symptoms (Shortness of breath [A], and Loss of taste / smell [B]) according to frequency experienced. For all symptoms assessed, HRQOL scores declined with increased frequency as experienced by the participant.

HRQOL = health-related quality of life; MCS = mental component score; PCS = physical component score.

## Supplementary Figure 2. Estimated Marginal Means of Physical and Mental Component Scores, and Utility Score for Cough (A) and Sore Throat (B)

**A. Cough**


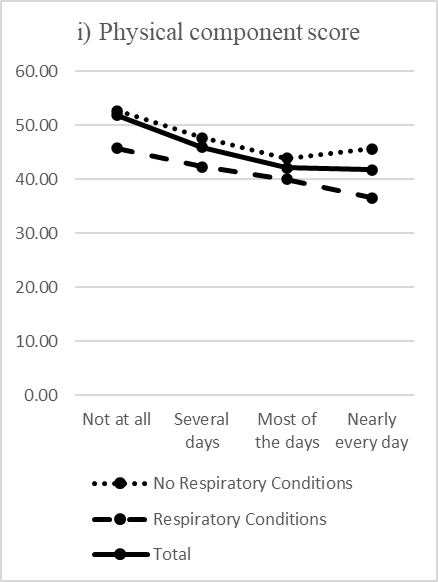

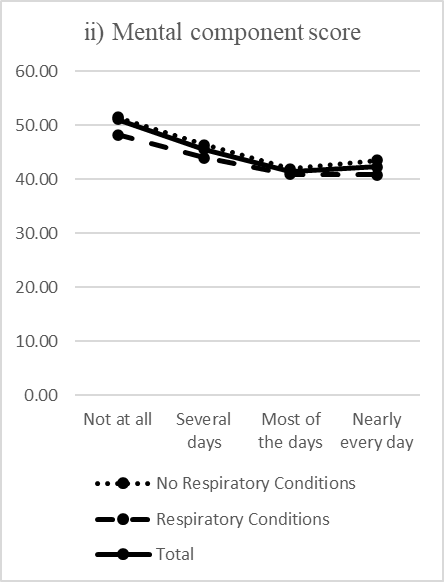

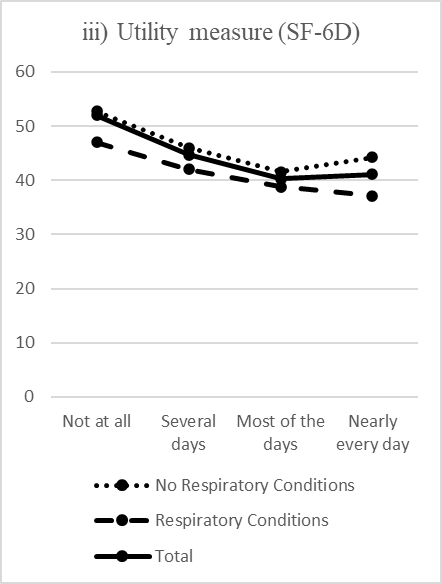


**B. Sore throat**


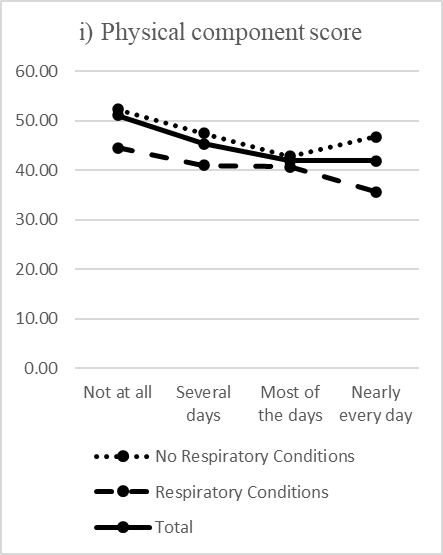

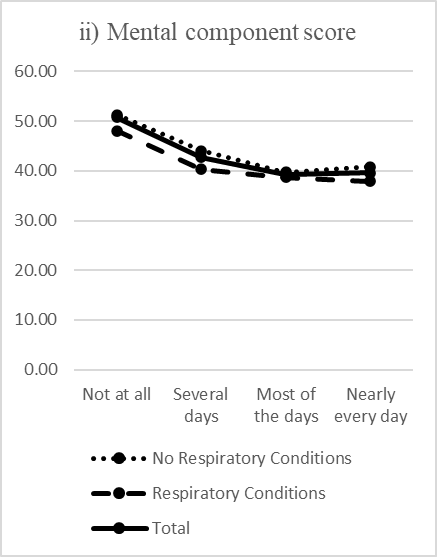

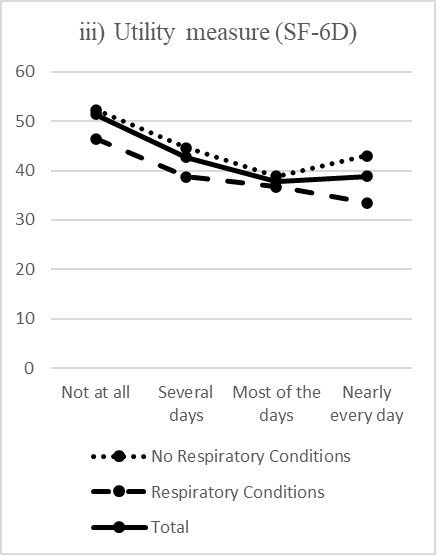


Supplementary Figure 2. Estimate marginal means of Physical (i) and Mental Component Scores (ii), and Utility (iii) for cough (A) and sore throat (B) with an interaction term for the category “nearly every day” and chronic respiratory conditions.

## Supplementary Figure 3. Estimated marginal means for the SF36v2 by symptom severity controlling for chronic respiratory conditions

**A. Loss of Taste and Smell**


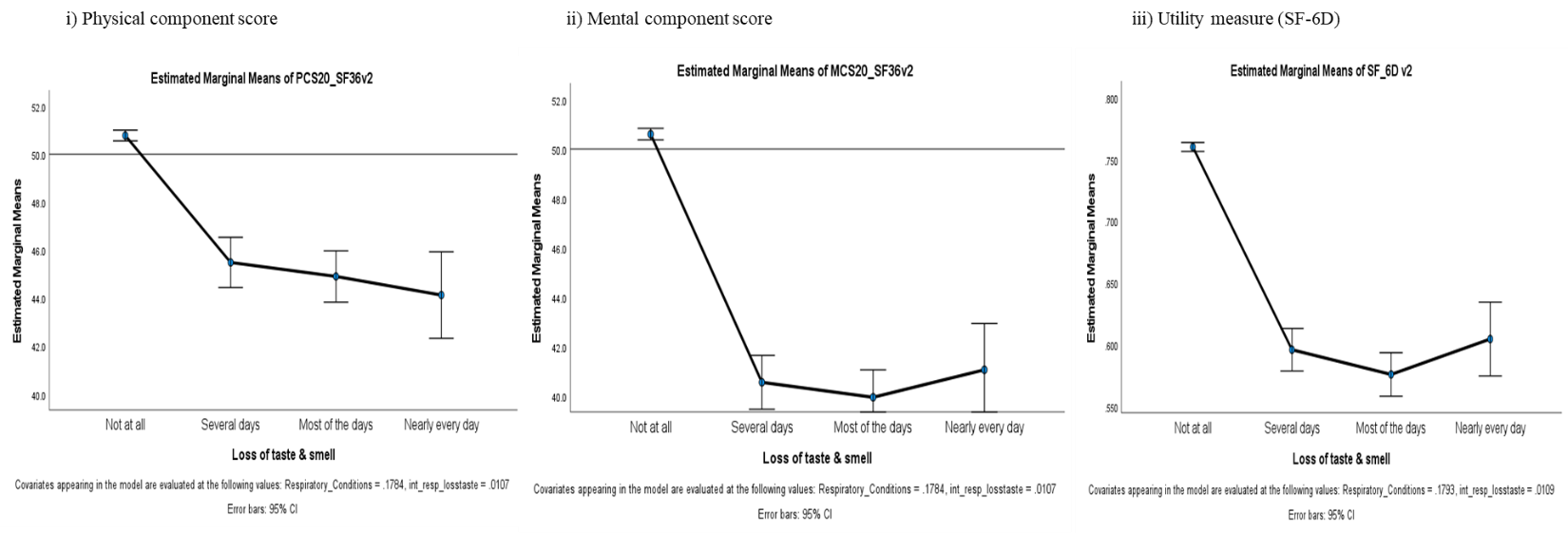


**B. Shortness of Breath**


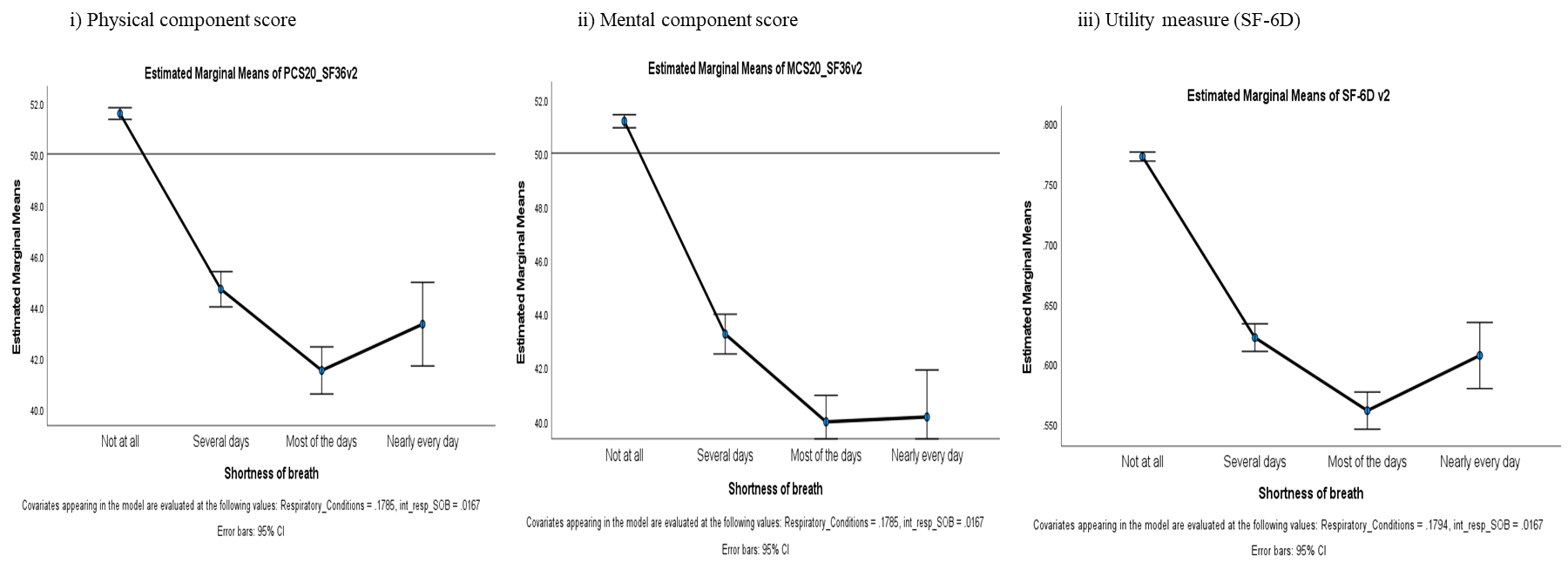


Supplementary Figure 3. Estimate marginal means of Physical (i) and Mental Component Scores (ii), and (Unstandardised) Utility measure (iii) for loss of taste and smell (A) and shortness of breath (B) with chronic respiratory conditions as a covariate.
